# Cooperative Tridentate Hydrogen-Bonding Interactions Enable Strong Underwater Adhesion

**DOI:** 10.1021/acsami.3c06545

**Published:** 2023-07-14

**Authors:** Zachary D. Lamberty, Ngon T. Tran, Christian D. van Engers, Preetika Karnal, Daniel B. Knorr, Joelle Frechette

**Affiliations:** † Chemical and Biomolecular Engineering Department, University of California, Berkeley, Berkeley, California 94760, United States; ‡ DEVCOM U.S. Army Research Laboratory, Aberdeen Proving Ground, Maryland 21005, United States; § Department of Chemical and Biomolecular Engineering, Johns Hopkins University, Baltimore, Maryland 21218, United States; ∥ Department of Chemical and Biomolecular Engineering, Lehigh University, 124 E Morton Street, Building 205, Bethlehem, Pennsylvania 18015, United States

**Keywords:** underwater adhesion, cooperative hydrogen bonding, epoxy, rate-dependent adhesion, surface forces apparatus, peeling, bond lifetime

## Abstract

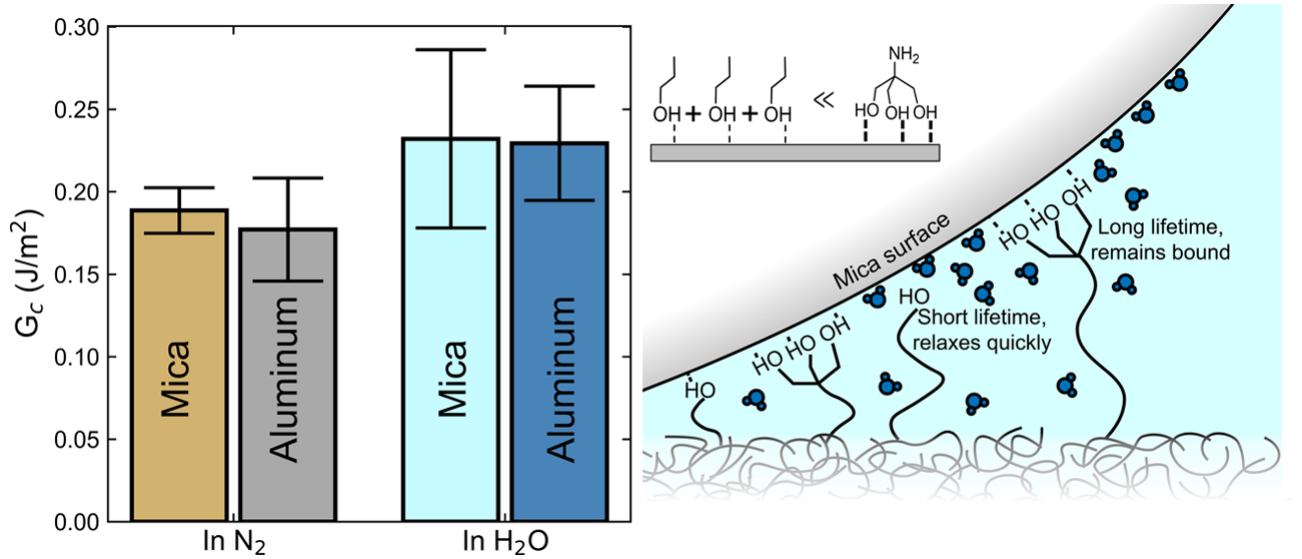

Multidentate hydrogen-bonding interactions are a promising strategy to improve underwater adhesion. Molecular and macroscale experiments have revealed an increase in underwater adhesion by incorporating multidentate H-bonding groups, but quantitatively relating the macroscale adhesive strength to cooperative hydrogen-bonding interactions remains challenging. Here, we investigate whether tridentate alcohol moieties incorporated in a model epoxy act cooperatively to enhance adhesion. We first demonstrate that incorporation of tridentate alcohol moieties leads to comparable adhesive strength with mica and aluminum in air and in water. We then show that the presence of tridentate groups leads to energy release rates that increase with an increase in crack velocity in air and in water, while materials lacking these groups do not display rate-dependent adhesion. We model the rate-dependent adhesion to estimate the activation energy of the interfacial bonds. Based on our data, we estimate the lifetime of these bonds to be between 2 ms and 6 s, corresponding to an equilibrium activation energy between 23*k*_B_*T* and 31*k*_B_*T*. These values are consistent with tridentate hydrogen bonding, suggesting that the three alcohol groups in the Tris moiety bond cooperatively form a robust adhesive interaction underwater.

## Introduction

Multidentate hydrogen-bonding moieties are promising functional groups for strong and water-resistant adhesives.^1^ Multidentate bonds are thought to be more stable than their monodentate counterparts, as multidentate interactions are kinetically and entropically favored due to the coordination between multiple adjacent binding groups.^2^ Well-studied multiple adjacent hydrogen-bonding groups in adhesion include catechols (Dopa)^3^ and ureido-pyrimidinone (UPy),^4^ both of which were proven to maintain strong interactions underwater. Multidentate hydrogen-bonding moieties also interact strongly with a wide range of surface chemistries.^5^ Single-molecule^6,7^ and macroscale^4,8^ measurements support the hypothesis that adjacent alcohol groups work cooperatively to stabilize and strengthen adhesive contact, helping to resist displacement by interfacial water.^9^ Yet, a quantitative relationship linking the cooperative bonding dynamics to strong macroscale adhesion remains elusive.^9^

**Figure 1:**
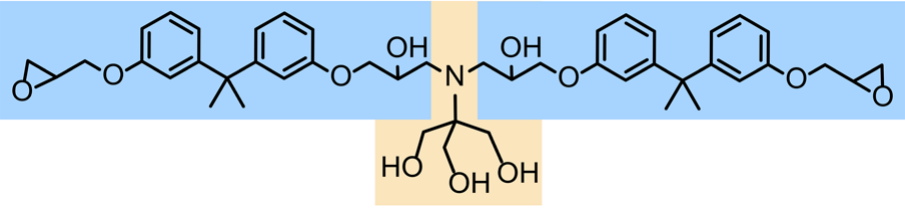
Oligomer structure. Structure of the Tris-modified DGEBA (DGEBA–Tris) oligomer. DGEBA sections are shaded in blue, and the Tris moiety is marked in yellow.

Recent work with the tri-alcohol molecule tris(hydroxymethyl)amino methane (Tris) shows the potential of these moieties for enabling strong underwater adhesion.^10,11^ These tridentate groups are particularly well-suited for epoxies as they can be readily reacted into the polymer backbone through the amine linkage. Stronger-bonded and water-tolerant epoxies are essential for structural adhesive and composite applications.^12−14^ Tran and co-workers showed that a simple surface pretreatment with Tris buffer improved dry and hot/wet aged lap shear strength of diglycidyl ether of bisphenol A (DGEBA) epoxy-bonded aluminum to a level comparable with their best polydopamine surface treatments.^11^ DGEBA is a well-studied and commonly used epoxy adhesive^10^ and thus serves as a suitable model backbone. Next, they incorporated Tris groups directly in the backbone of DGEBA (Tris–DGEBA, see Figure 1) and demonstrated lap shear strength after water aging that rivaled the strength of a silane pretreatment benchmark but without the need for the extensive surface pretreatment.^10^ Furthermore, they demonstrated that this improvement was not seen in epoxies functionalized with monodentate or bidentate alcohol groups, even when accounting for the hydroxyl concentration within the epoxy.^10^ These prior studies motivate the need to uncover the importance of cooperative hydrogen bonding in adhesion generally, and due to its industrial importance as a structural adhesive, of Tris-containing epoxies specifically.

Relating macroscopic adhesion measurements to intermolecular forces can be both experimentally and theoretically challenging, even in the limit where adhesive contact is caused by breaking interfacial bonds (and not bulk viscoelasticity). One approach that has been successful involves quantifying the rate dependence of crack propagation on the strength and stretch of chemical bonds in adhesive contact. The finite bond lifetime from chemical kinetics leads to a bond dissociation force that increases with the loading rate on an individual bond, described through the Bell–Evans model.^15^ Macroscopically, the process is averaged over many bonds throughout the contact region, leading to rate-dependent adhesion that scales with [ln(*u*)]^2^, where *u* is the stretching velocity of the interfacial bonds during crack propagation.^16^ Moreover, the equilibrium bond lifetime τ, which itself is related to the bond activation energy *E*_a_ through Eyring’s equation, leads to a transition from a rate-independent to a rate-dependent debonding force as the detachment velocity increases. By determining this characteristic transition velocity, it is then possible to determine τ (and thus *E*_a_) for bonds formed in adhesive contact. This general methodology has been employed to characterize the adhesion of covalent bonds in a PDMS-silanized glass interface,^16^ the role of electrostatic interactions in hydrogel–hydrogel adhesion,^17^ and highly entangled hydrogen-bonding networks between oxidized PDMS and silicon.^18,19^ In this work, we hypothesize that we can determine if cooperative hydrogen bonding is present in DGEBA–Tris by characterizing and modeling how adhesive strength depends on the rate of crack propagation.

Here, we investigate the mechanism by which the incorporation of Tris groups in the backbone of DGEBA epoxies improves underwater adhesion. We use a model epoxy adhesive consisting of two DGEBA groups joined by a single Tris moiety, DGEBA–Tris (Figure 1). We first report on the dependence of the strength of DGEBA–Tris/mica contact on the rate of crack propagation^20^ in air. We compare the adhesion of cured and uncured DGEBA–Tris to mica to other DGEBA epoxies that do not contain the Tris group. We then measure adhesion between thin films of DGEBA–Tris oligomers to mica in air and water using the surface forces apparatus (SFA).^21^ We further test the adhesion of DGEBA–Tris to aluminum in air and water to demonstrate the application to industrially relevant substrates. Finally, we model^16^ the dependence of the adhesive energy on the rate of crack propagation to obtain the threshold velocity above which adhesion is rate-dependent. From this critical velocity, we obtain estimates of interfacial bond lifetimes that we can use to test the hypothesis that cooperative hydrogen bonding is responsible for the strong adhesion in air and in water of DGEBA–Tris epoxies.

## Connecting Crack Propagation Velocity to Chemical Bond Kinetics

**Figure 2:**
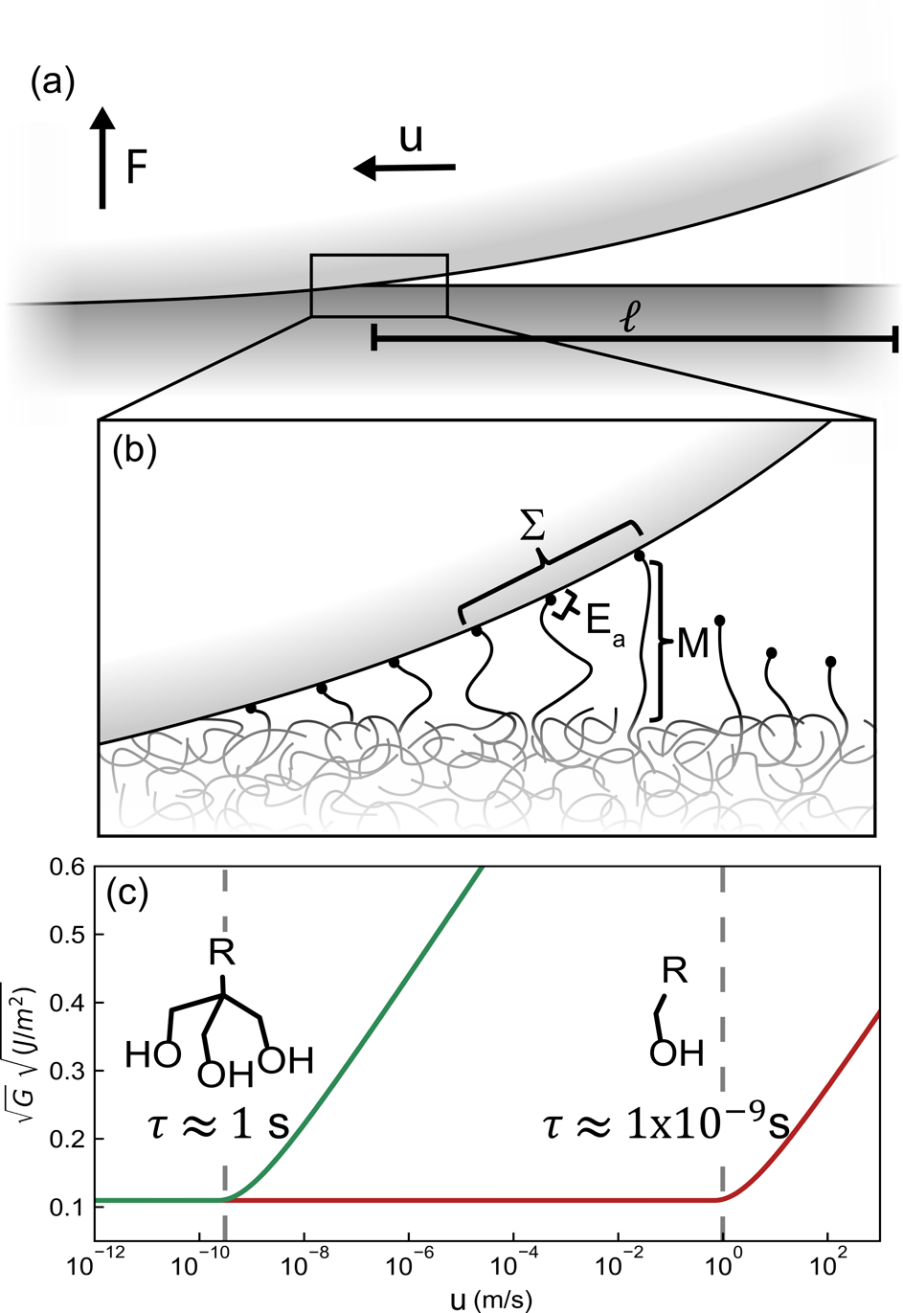
Relationship between interfacial bonds and adhesion. (a) Diagram of two adhesive surfaces in contact. *F* is the force on the two bodies,  is the length of the crack, and  is the velocity of the crack. (b) Enlarged schematic of the interface near the crack tip illustrating the interfacial bonds between the two surfaces. Σ is the surface density of bonds, *E*_a_ is the bond activation energy, and *M* is the polymer spring constant. (c) Predictions from eq 1 for the dependence of *G* vs crack velocity contrasting individual and multidentate hydrogen bonds. For τ = 1 s (green), the rate-dependent transition occurs at ∼0.5 nm/s, while for τ = 1 s (red), the transition occurs at ∼1 m/s.

Consider the interaction between two macroscopic surfaces, as illustrated in Figure 2a, where adhesion is dominated by bonds formed between the interacting surfaces in contact. A crack of length  exists between the two interacting bodies at the edge of the contact region. As a tensile force *F* is applied, the bodies are pulled apart and the crack propagates at the interface between the materials, while adhesive forces act to hold the bodies together and resist crack motion. The location of the crack is determined by the balance between elastic energy and the energy release rate *G*, which comprises interfacial bonds and dissipative phenomena that oppose the crack motion.^22^ If we zoom in around the crack tip (Figure 2b), individual chemical bonds of activation energy *E*_a_ and number density Σ act to hold the surfaces together. These bonds must be broken for the crack to advance. If each bond is attached to the bottom surface by a polymer of spring constant *M* and stretched at a velocity *V*_stretch_, the loading rate on each bond is . We approximate , where *u* is the velocity of the crack. The Bell–Evans theory tells us that the energy dissipated in breaking each bond depends on the loading rate,^15^ and many such bonds must be broken simultaneously to extend the crack. Chaudhury extended the Bell–Evans theory to macroscale contacts by summing up the energy dissipated during the breaking of a multitude of bonds to obtain the relationship given in eq 1 between *G* and the crack velocity^16^
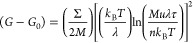
1In eq 1, *G*_0_ is the threshold (rate-independent) energy release rate, λ is a characteristic length scale for the bonds, and *n* is the number of bonds per chain. Note that eq 1 only applies in the limit where the  or when the thermal energy of the bonds is lower than the equilibrium energy barrier for bond dissociation.^23^ In the slow crack velocity limit (, the thermal energy dominates, and the energy release rate is by definition *G* = *G*_0_. Finally, the lifetime of a bond τ in eq 1 can be estimated using Eyring’s equation^16^
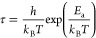
2where *h* is Planck’s constant, *k*_B_ is Boltzmann’s constant, and *T* is the temperature. Recently, more rigorous models have been developed that are in qualitative agreement with eq 1, but require additional details for their implementation.^24^ While eq 1 can be applied to any type of interfacial bond, the onset of rate dependence is highly dependent on τ and, by extension, *E*_a_. For an individual hydrogen bond, *E*_a_ ≈ 10*k*_B_*T*.^17,25^ As a first-order approximation, for cooperative hydrogen bonds, we expect *E*_a_ to scale with the number of hydrogen bonds, *N*, acting cooperatively such that *E*_a_ ≈ N*10*k*_B_*T*. In the case of Tris and tridentate bonds, *N* = 3 and *E*_a_ ≈ 30*k*_B_*T*, which leads to a bond lifetime of τ ≈ 1 s and the onset of rate dependence of adhesion at a threshold velocity of *u* ∼ 0.5 nm/s. Therefore, we expect rate-dependent behavior in the nm/s regime to correspond to cooperative tridentate hydrogen bonding. In contrast, for monodentate hydrogen bonding, the threshold velocity would be *u* ∼ 1 m/s. This rate dependence should occur in both air and in water if cooperative bonds can be formed but should be absent in epoxy analogs that lack Tris groups. Figure 2c demonstrates the expected dependence of *G* with the crack velocity obtained from eq 1 for two nominally identical materials (same number of sites and polymer spring constant). While the threshold velocity occurs at ∼0.5 nm/s for tridentate hydrogen bonds (*E*_a_ = 30*k*_B_*T* and τ ≈ 1 s), this transition would not occur until 1 m/s for a monodentate bond (*E*_a_ = 10*k*_B_*T* and τ ≈ 10^–9^ s). For all but the fastest measurements, adhesion from single hydrogen bonds will be rate-independent.

## Materials and Methods

### Materials

HNO_3_ (68%, BDH), ethanol (200 proof, Pharmco), H_2_O_2_ (30%, Fisher), H_2_SO_4_ (95.0%, J.T.Baker), tetrahydrofuran (THF, >99.5%, Supelco), ethyl acetate (99.9%, Fisher), muscovite mica (Ruby, ASTM V-1/V-2, S&J Trading), silver pellets (99.999%, Alfa Aesar), aluminum pellets (99.999%, Kurt J. Lesker), DGEBA (Hexion Inc. EPON Resin 825), EPON Resin 1004 F (Hexion Inc.), and 2-amino-2-(hydroxymethyl)-1,3-propanediol (Tris, >99.9%, Sigma-Aldrich) were purchased and used as received. Deionized (DI) water (>18.2 MΩ cm resistivity) was obtained from an EMD Milli-Q Integral Water Purification System.

### Synthesis of DGEBA–Tris Oligomers

2-(Bis(1-hydroxy-2-(4-(2-(4-(oxiran-2-ylmethoxy)phenyl)propan-2-yl)phenoxy)ethyl)amino)-2-(hydroxymethyl)propane-1,3-diol diglycidyl ether of bisphenol A–tris(hydroxymethyl)amino methane, DGEBA–Tris) oligomers were synthesized through nucleophilic epoxide ring opening of the epoxy DGEBA oligomers by Tris amine groups, as reported previously.^10^ In brief, DGEBA monomers and Tris (2:1 stoichiometric ratio) were dissolved in ethanol (5.6 M DGEBA, 2.8 M Tris) at 78 °C under constant stirring for 18 h to allow the Tris molecules to bind two DGEBA groups through the amine linkage, forming the DGEBA–Tris oligomer. Afterward, the oligomers were diluted to 180 mM in ethanol for storage.

### Preparation of Self-Arresting Crack Samples

Large muscovite mica sheets (∼2 cm × 6 cm, 10–30 μm thickness) were cleaved in a laminar flow cabinet and cut into rectangles comprising mostly of a single crystal. The sheets were then rinsed with 5 mL of an 8μ L HNO_3_/50 mL H_2_O solution to exchange K^+^ ions from the mica surface with H^+^ and then blow-dried with N_2_. Oligomer or polymer solutions were created that had an equal concentration of 34 mM DGEBA units in THF (e.g., 17 mM DGEBA–Tris with 2 DGEBA units per oligomer). Then, 500 μ L of solution was spin-coated onto the HNO_3_-treated mica sheets, followed by heating for 1–2 h at 100 °C under vacuum to remove residual solvent. Meanwhile, thick mica base sheets (∼150 cm^2^ area, ∼1 mm thick) were freshly cleaved and rinsed with 20 mL of HNO_3_ solution before drying as before. After the oven-drying was complete, the oligomer-/polymer-coated mica sheets were placed polymer-side down on the mica base sheets and the two sheets were firmly pressed together. Multiple epoxy-coated sheets were affixed to the same base sheet and then cut apart to size. A steel block was placed on each sample to provide additional weight during annealing in an oven for 1 h at 80 °C under vacuum. Afterward, uncured samples were removed to be tested, while samples to be cured remained in the oven as the temperature was ramped over a period of 2 h to 150 °C under a gentle flow of N_2_, followed by curing at 150 °C for the requisite curing time (6 or 18 h).

### Preparation of Surfaces for SFA Experiments

The preparation of mica surfaces is described in detail in our prior work.^26^ Details are mentioned here for clarity. Single-crystal muscovite mica sheets (3–8 μm) were cleaved in a laminar flow cabinet and placed on a larger mica backing sheet. A thin layer of silver (∼50 nm) was then thermally evaporated onto the exposed side of the mica sheets (using a Kurt J. Lesker Nano 38 thermal evaporator). The mica surfaces were then glued to the SFA disks using a uniform layer of EPON epoxy (∼5 μm) deposited onto the hemi-cylindrical quartz lenses (radii of curvature ∼ 2 cm). To obtain a uniform EPON layer, the glue was first dissolved in an ethyl acetate solution that was then spin-coated on a disk. After spin-coating, the glue was melted by heating the lenses on a hotplate at 225 °C and a cleaved sheet of mica was placed silver-side down on the lens. After cooling, the mica lenses were immersed in a weak HNO_3_ solution (8 μL HNO_3_/100 mL H_2_O) to exchange K^+^ ions from the mica surface with H^+^. In all SFA experiments, one of the mica surfaces was coated with the DGEBA–Tris oligomer. For oligomer-coated lenses, a solution of 17 mM DGEBA–Tris oligomer in ethanol was spin-coated onto the H^+^ mica lenses and then annealed in a vacuum oven at 80 °C for 6 h to remove excess solvent. Ultra-smooth aluminum surfaces were fabricated according to our previously published procedure^26^ by templating thermally evaporated aluminum films with mica and then removing the mica template in water.

### Atomic Force Microscopy Imaging

Atomic force microscopy (AFM) data were recorded using a Bruker Dimension 3100 atomic force microscope (Bruker Nano, Santa Barbara, CA) in tapping mode and analyzed using Gwyddion 2.52. For processing the AFM data, we align the line profiles using a first-degree polynomial (linear slope) and subtract the background using a third-degree polynomial for both the *x*- and *y*-directions. Root-mean-square (rms) roughness values were calculated over a 5 μm × 5 μm area using Gwyddion’s built-in statistical functions. AFM imaging was performed on both as-prepared (dry) samples and samples that had been soaked in DI water overnight, to reproduce the conditions in SFA experiments.

### Ellipsometry

Ellipsometry measurements were performed with an Accurion Nanofilm EP3 single-wavelength (532 nm) variable-angle imaging ellipsometer with a 20× objective. DGEBA–Tris oligomer films were deposited onto clean silicon wafers using the same procedure as for SFA samples, except for the K^+^ ion exchange step. We assume that the oligomer thickness is (on average) identical to that of the films deposited on the mica sheet for SFA experiments. For each sample, first, the angle of incidence was varied to find the Brewster’s angle and then the reflectance of the sample was measured at angles around the Brewster’s angle. Accurion’s EP4 software was then used to analyze the measured data, and both the refractive index and thickness of oligomer films were varied to fit the reflectance data. A film thickness of 115.4 ± 1.5 nm with a refractive index of *n*_D_ = 1.582 was obtained from ellipsometry. The refractive index value for the DGEBA–Tris oligomer was then used for the analysis of the SFA interferometric data.

### Surface Tension

Surface tension measurements were conducted with a Dataphysics OCA 15EC contact angle goniometer. The fluids investigated were 18.2 MΩ cm H_2_O that was either in contact with DGEBA–Tris films or only in a glass beaker (control) for 24 h. A 25 μL drop was hung from a clean stainless-steel needle with an OD of 0.51 mm. Measurements were performed in laboratory air. Images were taken continuously over 100 s, during which no noticeable change in surface tension occurred. Image analysis and surface tension calculations were performed by Dataphysics’ SCA software.

### Adhesion Measurements through Self-Arresting Crack Propagation

Peeling by crack propagation and arrest was performed with a procedure inspired by previously reported methods^20,27^ on a modified Zeiss Axiovert 135 inverted microscope equipped with a translating stage. The method relies on peeling apart two adhered surfaces by bending the upper (more flexible) sheet and monitoring the subsequent propagation of the peeling front (crack). The crack will advance and eventually arrest once mechanical equilibrium is reached between the elastic bending of the sheet and the adhesive forces resisting separation. All the self-arresting crack measurements were conducted between two mica base sheets adhered together with a thin layer of epoxy prepared as described above. Adhesion measurements started by prying apart the two mica sheets at one end with a needle, followed by inserting a 1 mm diameter glass rod as a spacer in the gap created by the needle. The glass rod creates a crack at the epoxy–mica interface that grows and arrests once equilibrium between the bending and surface forces is achieved. Then, we advanced the glass rod and monitor the associated crack propagation. As the glass rod moved to peel the mica sheet, the crack length was imaged using a 5× objective and a 555–565 nm band-pass filter to observe the interference patterns produced by the crack opening. Acquisition time began at 10 fps for each measurement but gradually slowed to 1 frame per 5 min over the course of an experiment as the rate of crack propagation slows down. To quantify the crack length, the edge of the crack was assumed to be approximately the location of the first visible constructive interference fringe. For each measurement, a location ∼3 mm from the previous crack front was located with the camera and then the spacer was rapidly pushed by 3 mm to move the crack into view. Each time the glass rod was moved to advance the crack, the crack length was measured for several hours as it advanced and returned to its steady-state length. This process was repeated several times on each sample by continuing to advance the crack further. The crack velocity was calculated by first smoothing the raw crack length as a function of time over using a first-degree Savitzky–Golay filter and then interpolating the data to a continuous function to eliminate the effects of the variable sampling rate. Dividing the interpolated function into 1000 logarithmically spaced points then allows for fitting crack position vs time to a line using the 60 nearest neighbor points in time and calculation of the instantaneous crack velocity as the fitted slope. The thicknesses of the mica top sheets are measured through multiple-beam interferometry^28^ by evaporating ∼50 nm films of Ag onto both sides. Measurements were repeated at least five times on each sample and with at least two separate samples.

### SFA Measurements

Surface force measurements were performed with an SFA 2000 (SurForce LLC, Supporting Information Figure S1) with an Andor Shamrock spectrometer and CCD camera (Andor Zyla 5.5 sCMOS). Technical aspects of the SFA are given elsewhere, and only the aspects specific to our experiments are described here.^26^ SFA measurements were performed between disks arranged in a cross-cylindrical configuration, which is equivalent to a sphere-plane geometry. One of the surfaces is mica, and the other is mica coated with a DGEBA–Tris oligomer. The separation between the surfaces was obtained using multiple-beam interferometry.^28^ Spectral data are converted into surface separation using the fast spectral correlation algorithm.^26,29^ A microstepping motor was used to control the motion of the lower surface, which was mounted on a cantilever spring with a spring constant of 2238 ± 44 N/m. The optical constants of the mica and silver were taken from the literature,^30,31^ while the refractive index of the DGEBA–Tris oligomer was measured using ellipsometry. Mica thickness was measured by conducting separate calibration experiments with mica pieces of the same thickness. Because of reswelling and minor stretching in the film during retraction, the film thickness changes when the surfaces are in contact with an applied load (tension or compression). Therefore, to determine the contact radius, we converted the shape of interference fringes at any given time and extract the edge of the flattened region of the profile. The edge of contact is defined by the slope of the profile, with any slope below 0.5 nm vertical change per 1 μm horizontal change in the profile designating contact between the surfaces. The radial velocity *u*_r_ was then found by analyzing the rate of change of contact radius over time. The instantaneous value of *u*_r_ was estimated by calculating the slope of *a* vs *t* for the 10 nearest neighbor points at the time of interest.

After assembling the disks in the SFA, the system was allowed to equilibrate under a gentle flow of dry nitrogen for at least 1 h with all equipment running prior to any measurements. For each adhesion measurement, the surfaces were brought into contact quasi-statically using individual steps with a drive velocity of ∼28 nm/s for 0.5 s followed by a 4.5 s pause. Once contact between the surfaces was made (*D* = 0, visually seen as a sudden slowing of fringe movement), the samples were compressed at the same quasi-static velocity for an additional 800 s to reach a dwell force of 5 mN (*F*/*R* ∼ 0.3 N/m). Afterward, the surfaces relaxed while in contact for a dwell time of 5–20 min. After dwell, a constant pre-determined motor velocity (between 5 and 75 nm/s) was used to separate the surfaces until detachment (jump out). For each experiment, adhesion between the DGEBA–Tris oligomer-coated surface and mica was first measured in air twice before adding water. Water was then introduced by injecting 50 μL of DI water between the surfaces to form a capillary meniscus. To reduce the effect of evaporation, an additional 3 mL of water was injected into the bottom of the SFA chamber. After injection, the samples were left to equilibrate in water for at least 1 h before further measurements. Depending on the motor velocity, it took between 5 and 30 min to separate the surfaces. After each contact, the radii of curvature of the surfaces were measured at two orthogonal orientations and the geometrical mean was reported, . Then, the relative position of the two disks was changed to be able to repeat the measurements on a new spot. Three different samples were investigated, with 8–17 independent spots per sample. Motor velocity was calculated for each retraction by calibrating the sample movement to the applied voltage on the motor when the samples reached a force-free regime.

## Results and Discussion

### Characterization of the DGEBA–Tris Oligomer Films

**Figure 3:**
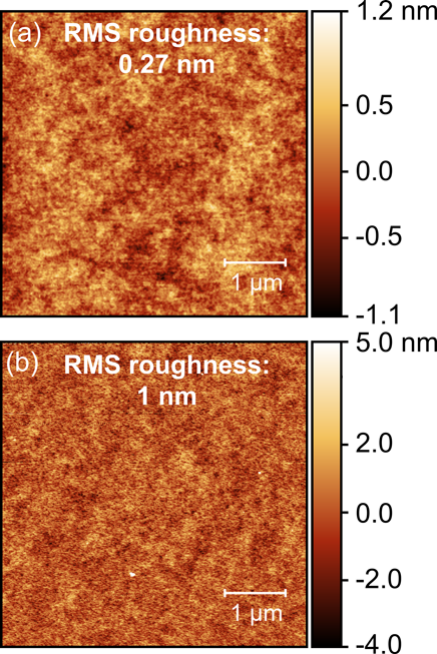
Characterization of DGEBA–Tris using AFM. (a) AFM image of a representative area on the surface of an as-deposited DGEBA–Tris film, showing an rms roughness of 0.27 nm. (b) AFM image of the surface of a DGEBA–Tris film that has been soaked in water overnight. The surface is featureless with a roughness of 1.0 nm rms.

We characterized the surface roughness of the DGEBA–Tris films using AFM after the annealing step (as-deposited), as well as after the samples were submerged in water overnight (Figure 3). AFM imaging of the annealed films reveals a featureless surface with a low rms roughness of 0.27 nm (2.21 nm peak-to-valley) over a 1 μm × 1 μm area. Similarly, imaging of DGEBA–Tris films that were soaked in DI water overnight showed a surface that remains smooth and featureless but with an rms roughness of 1 nm (9.23 nm peak-to-valley). The increase in rms roughness is consistent with the small degree of swelling observed in SFA experiments.

Bulk DGEBA–Tris films are insoluble in water. We also investigated if some small amount of DGEBA–Tris might dissolve in water and become surface active, which in turn could affect adhesion. To do so, we immersed 4 cm × 4 cm sheets of mica coated with the DGEBA–Tris oligomer films in a small amount of DI water for 24 h at room temperature. We then measured the surface tension of the water using a pendant drop tensiometer and obtained 72.5 ± 0.08 mN/m, indistinguishable from that of clean water.

### Tridentate and Monodentate Adhesion in Air

To determine if cooperative bonding is important in the adhesion of DGEBA–Tris, we compare its adhesion with mica to analogs without the Tris moiety. The first control is the primarily monomeric DGEBA of an average molecular weight (MW) of 355 g/mol, hereafter referred to as DGEBA-355. Since DGEBA-355 is a liquid at room temperature, it is cured for 18 h at 150 °C to form a solid film. The second control is a glassy DGEBA polymer with an average of 5.5–6 repeat units per chain; this material has an average MW of 1750 g/mol and is referred to as DGEBA-1750. DGEBA-1750 samples were also further cured at 150 °C for 18 h. The last control is a test of the experimental protocol itself; here, we do not employ any adhesive and characterize the interfacial crack propagation during the separation of two mica sheets (no intervening oligomer or polymer layers). As none of these materials contain Tris groups, we do not anticipate them to exhibit rate-dependent adhesion.

Our primary tridentate material is oligomeric DGEBA–Tris (Figure 1), where each molecule contains exactly one Tris group. Oligomeric DGEBA–Tris is a glassy solid with a *T*_g_ of 31.7 °C. To better compare with our control materials, we also investigate DGEBA–Tris that has been cured at 150 °C for 18 h. Curing DGEBA–Tris will also increase the average chain length, leading to a higher polymer spring constant.

**Figure 4:**
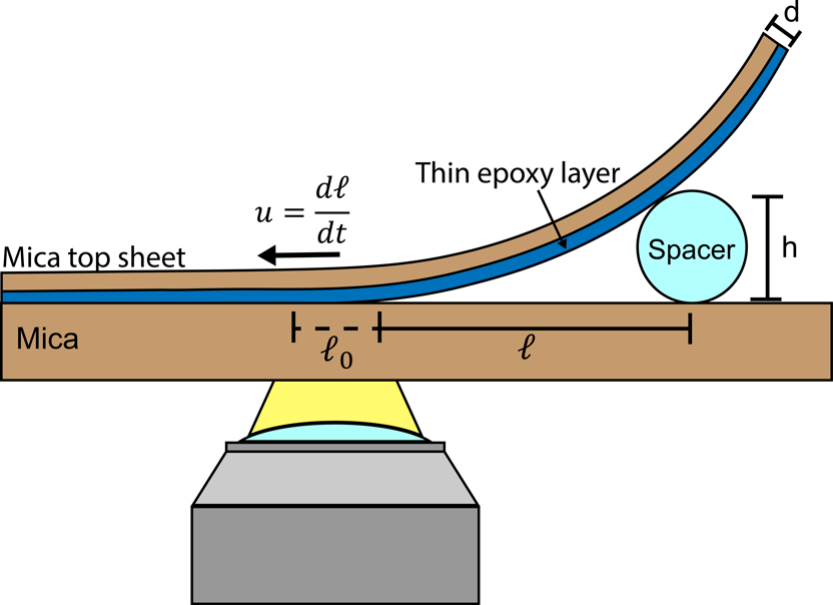
Diagram of interfacial crack propagation measurements. Schematic of the geometrical configuration for interfacial crack measurements, where  is the crack length, *h* is the height of the spacer, and *d* is the thickness of the mica top sheet. Note that the thin epoxy layer (blue) is ∼100 nm thick and is extremely thin relative to the mica top sheet thickness (10–30 μm) (not to scale).

**Figure 5:**
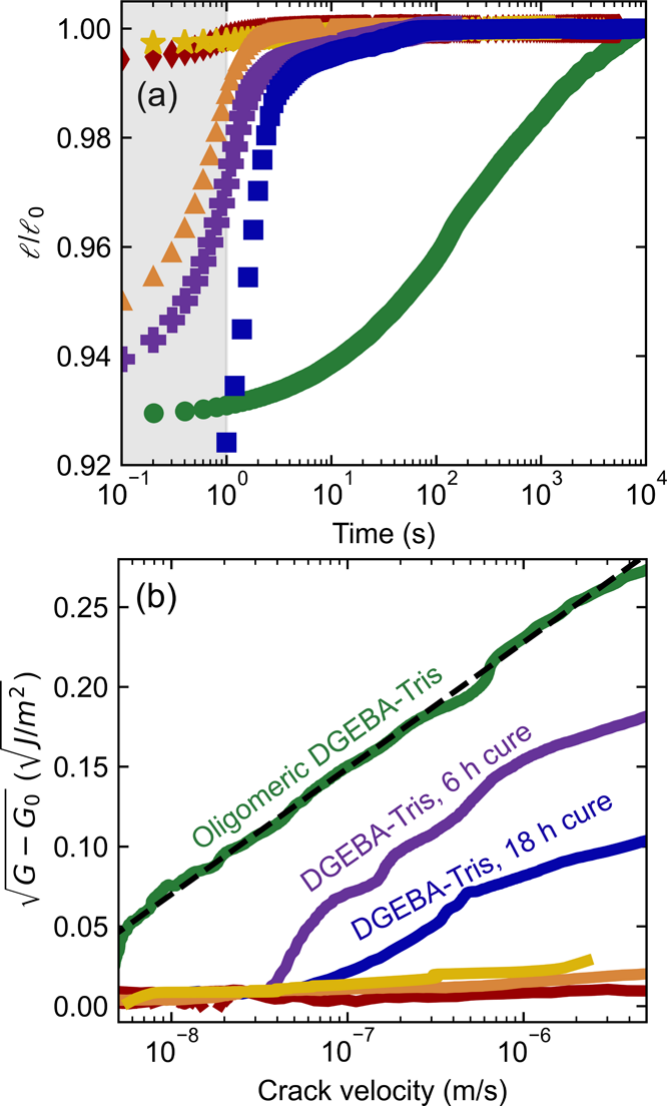
Crack growth and adhesion dynamics. The materials shown are bare H^+^ mica (orange triangles), oligomeric DGEBA–Tris (green circles), DGEBA–Tris cured for 6 h (purple crosses) or 18 h at 150 °C (blue squares), and DGEBA-355 (yellow stars) and DGEBA-1750 (red diamonds), both cured for 18 h at 150 °C. The opposing surface for all materials is a thick H^+^ mica sheet. (a) Crack length normalized by the final measured crack length value (, dashed line) as a function of time after the movement of the spacer. The crack propagation in the first second (shaded region) is dominated by air resistance. (b) Scaled rate-enhanced adhesion vs crack velocity on a logarithmic scale. The black dotted line shows fit to oligomeric DGEBA–Tris data (*R*^2^ = 0.997).

For all experiments, the thin mica top sheet is coated with a thin (∼100 nm) oligomer/polymer film and then bonded to a thick mica base sheet (Figure 4). We propagate a crack through the epoxy/mica base sheet interface and monitor the crack growth and velocity over time as the crack returns to its equilibrium length . We choose to use self-arresting crack propagation to characterize the probe adhesion across orders of magnitude in crack velocities in a single experiment. Representative crack length versus time curves for each epoxy is shown in Figure 5a. When peeling mica from mica, DGEBA-355, and DGEBA-1750, the crack rapidly extends and reaches its equilibrium length within 1–2 s. That first second of motion, indicated by the gray shaded area, is dominated by dynamic effects including air resistance, but afterward, the crack length remains nearly constant. In contrast, for oligomeric DGEBA–Tris samples, we observe continual crack motion over several hours, which gradually slows down as the crack nears its final length. Similar long-term crack movement is observed for cured DGEBA–Tris samples, although extension halts after about an hour.

The length of the crack is controlled by the force balance between the bending moment on the mica top sheet and the adhesive forces holding the materials together. For rigid materials, this force balance can be expressed by the equation^20^
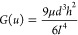
3Here, μ is the shear modulus of the top mica sheet (μ = 25 GPa, Poisson ratio ν = 0.21),^32^*d* is the thickness of the top mica sheet (measured independently, ∼10–30 μm), and *h* is the fixed height of the spacer. We then used the measured crack length as a function of time to calculate the crack velocity  and the corresponding *G*(*u*) for each sample. Once we reach a crack velocity of <1 nm/s, we consider the crack to have stopped and measure the final value of  at least 1 h after this point. The value of  is taken to correspond to *G*_0_, the threshold energy release rate. For most samples, the crack stops within 15 min. However, oligomeric DGEBA–Tris samples required us to wait longer (several hours) to reach an equilibrium crack length, and even then, small changes in the crack length continued for at least one day.

We investigated if the energy release rate increases with crack velocity and if the rate dependence for DGEBA–Tris follows the scaling expected for the breaking of interfacial bonds described by eq 1. Specifically, eq 1 predicts a linear relationship between  and ln(*u*). As illustrated by Figure 5b, oligomeric DGEBA–Tris films have a strong enhancement in adhesion with crack velocity, for a crack velocity above 5 nm/s. Moreover, the increase in energy release rate with crack velocity follows the scaling predicted by eq 1. Similarly, adhesion of cured DGEBA–Tris films also increases with crack velocity but only for *u* > 50 nm/s. Cured DGEBA–Tris films reliably show an increase in the onset velocity for adhesion-enhancement but have higher variability than oligomeric DGEBA–Tris samples (Supporting Information Figure S2). In contrast, the adhesion of mica, DGEBA-355, and DGEBA-1750 does not increase with an increase in crack velocity after the first second of motion (<10 μm/s).

The fact that adhesion of DGEBA–Tris films increases with crack velocity but none of the controls do strongly suggests that the increase in the energy release rate is due to the presence of Tris groups. Moreover, the linear relationship between  and , as predicted by eq 1, supports the hypothesis that the increase in adhesion is due to the breaking of interfacial bonds. Finally, the shift to higher threshold velocity between oligomeric and cured DGEBA–Tris suggests an increase in the effective polymer spring constant, *M*, with curing.^33^

We extract an estimate of the interfacial bond lifetime, τ, from the dependence of the energy release rate on the crack velocity for oligomeric DGEBA–Tris using eq 1. In addition to the bond lifetime, the spring constant of the oligomer (*M*) and the surface bond density (Σ) are also unknown, but only two of the three parameters can be obtained independently from eq 1. All the other constants are known and displayed in Table 1. Once Σ and *M* are known, we can obtain τ from the intercept of the data shown in Figure 5b (*R*^2^ is between 0.96 and 0.997 for each curve). Fortunately, an upper limit on Σ and a lower limit on *M* will bound τ. To obtain a lower bound for *M*, we estimate the force–extension relationship of a single DGEBA–Tris oligomer using the modified freely jointed chain model combined with literature values for similar materials (see Supporting Information Figure S3).^34−38^ The stretching caused by entropic forces is a lower bound, with *M*_entropic_ ≥ 0.008 N/m. The upper bound would be a high extension limit dominated by the segment elasticity of the carbon backbone, giving *M*_elastic_ = 5 N/m. While these two numerical values are limiting cases, we suspect that the majority of the energy stored in the chain occurs in the high extension regime. To determine an upper bound for Σ, we rely on the known structure of mica. For the upper bound, we can assume that all the surface oxide groups on mica are taken by the −OH in the DEGBA–Tris oligomer, leading to a density of ∼1.4 × 10^19^ #/m^2^.^39−41^ The lower theoretical bound for the number density of bonds with the mica surface is zero.

**Table 1:** Parameters Used in Equation 1 and Values Obtained from Fitting the Data in Figures 5b and 9

	*T* (°C)	λ (nm)	*G*_0_ (J/m^2^)		Σ (#/m^2^)	*M* (mN/m)	τ (s)	*E*_a_ (*k*_B_*T*)
Dry	20	0.18^a^	0.23 ± 0.04	Bounding limits	≤1.4 × 10^19^^b^	≥8^c^	0.002–0.6	23–29
				Lake–Thomas	5.0 × 10^17^	73.0 ± 0.8^d^	0.060 ± 0.002	26.6 ± 0.03
Underwater	20	0.18^a^	0.012^e^	Bounding limits	≤1.4 × 10^19^^b^	≥8^c^	0.08–6	27–31
				Lake–Thomas	5.0 × 10^17^	19 ± 4^d^	3 ± 1	30.3 ± 0.6

a From refs (25)(42), and (43).

b From refs (39)–^41^.

c From refs (34)–^38^.

d Fitted.

e Calculated in Supporting Information Section 5.

Based on the upper limit on Σ and the lower limit on *M*, the bond lifetime is between 0.002 s ≤ τ ≤ 0.6 s. A bond lifetime on the order of milliseconds to seconds is orders of magnitude higher than what would be expected for individual hydrogen bonds. As a comparison, the lifetime of a single hydrogen bond is estimated to be *O*(ps – ns).^9,44^ The long bond lifetime is consistent with cooperative hydrogen bonding. Using Eyring’s equation (eq 2), the equilibrium activation energy of the bond is estimated to be 23*k*_B_*T* ≤ *E*_a_ ≤ 29*k*_B_*T*. We refine these bounds further using the Lake–Thomas theory to obtain an estimate of Σ ≈ 5.0 × 10^17^ #/m^2^ (see Supporting Information Section 4).^45^ Using the Lake–Thomas value for Σ gives τ = 0.060 ± 0.002 s and *E*_a_ = 26.6 ± 0.03*k*_B_*T*, with error bounds calculated through the standard error of the data.

To put these values for *E*_a_ in context, if the three Tris alcohols acted independently of each other during debonding, the number of bonds per unit area would increase threefold as a single Tris group will have three individual bonds with the mica surface. However, the lifetime would be that of a single hydrogen bond, of *O*(ns), corresponding to activation energies of ∼10*k*_B_*T*. In contrast, if the three hydroxyls act cooperatively and must debond from the surface at once, a simple addition of the individual activation energies for each −OH group will give *E*_a_ = 30*k*_B_*T* corresponding to τ = 1.7 s. These estimates for the simultaneous (cooperative) detachment of three hydrogen bonds are very close to the values we obtained in our experiments.

### Underwater Adhesion Measurements

**Figure 6:**
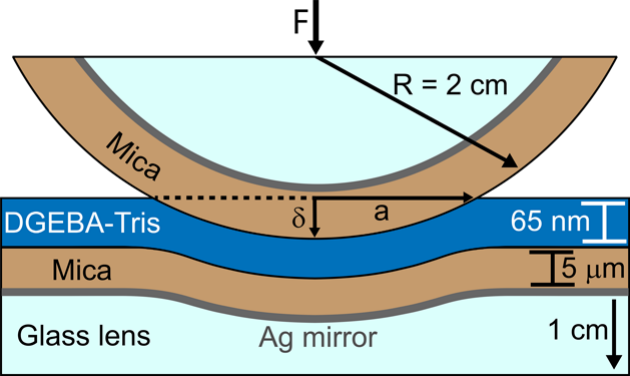
Diagram of SFA configuration. Schematic of the geometrical configuration for SFA adhesion measurements, where *F* is the force between the disks, *a* is the contact radius, and δ is the indentation depth. Not to scale.

We then characterize the adhesion of films of DGEBA–Tris oligomer in water using the SFA. The geometry of these measurements approximates a sphere-on-flat contact, as shown in Figure 6. With this technique, we simultaneously measure the surface separation *D* (or indentation depth δ), contact radius *a*, crack velocity (*u*), and force *F*. Force values are normalized by the radius of curvature *R* to obtain an interaction energy. The geometry at the crack tip is analogous to the one in the self-arrested crack measurements. We focus on interactions between oligomeric DGEBA–Tris and mica or aluminum due to the pronounced increase in adhesion with crack velocity measured in air. In contrast to previous work with DGEBA–Tris that studied the water-induced degradation of adhesive bonds that were formed in air,^10^ here we both form and break the adhesive bonds underwater.

**Figure 7:**
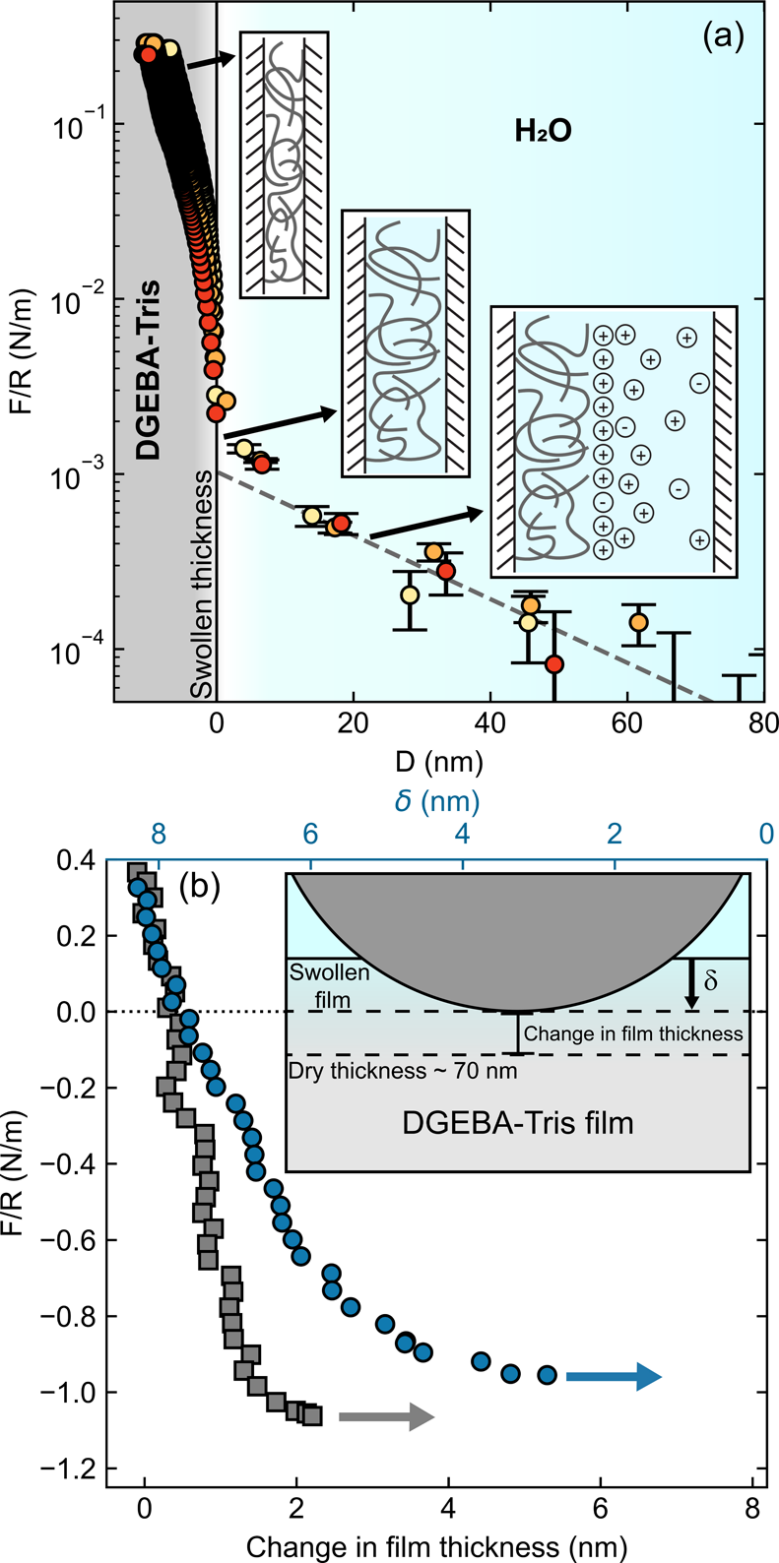
Forces during approach and retraction in adhesion measurements. (a) Measured force (normalized by the radius of curvature) vs surface separation *D* for three different samples approaching contact in water. Zero separation is set at the onset of the steric repulsion. Cartoons indicate the processes that occur during the approach, from right to left: electrostatic double-layer repulsion; contact with swollen film; deswelling and elastic compression of film. (b) Retraction force (normalized by the radius of curvature) vs change in film thickness in dry nitrogen (gray squares) and water (blue circles) at comparable velocities (23–26 nm/s). The top *x*-axis is added to show the underwater film indentation depth (δ) for swollen films (blue circles only), where zero is defined as the swollen film thickness as was done in the *x*-axis in (a).

We first characterize the interactions between the DGEBA–Tris film and mica as the surfaces approach and make contact. For samples in a dry N_2_ atmosphere, no forces are measured until attractive forces cause a spring instability and jump into contact. In contact, the DGEBA–Tris film thickness is measured to be 61–75 nm, the variability coming from different experiments. During the approach in water, we first observe a long-range repulsive force starting at ∼80 nm away from contact (Figure 7a), attributable to electrostatic double-layer repulsion (see Supporting Information Section 5).^46^ This is followed by a steep repulsion, which we ascribe to contact (and compression) with the swollen DGEBA–Tris film. The surfaces are compressed until we reach the set point of *F*/*R* = 0.3 N/m (∼5 mN), and use the separation at this set point to define contact in water (zero separation). By convention, we assign positive signs for compressive forces and negative signs for tension (adhesion). In water, the first contact with the swollen DGEBA–Tris occurs 5–15 nm away from contact in dry N_2_. Based on these values and the thickness of the dry films obtained from interferometry measurements before water was added to the system, we estimate the degree of swelling in water to be 17 ± 10%. Once the force set point of 0.3 N/m is reached, the films return to their initial dry thickness. We attribute this change in film thickness to the removal of most of the water in the film within the contact region during compression. Prior to detachment, the surfaces are kept at *F*/*R* = 0.3 N/m for 5 min. During this dwell period, the thickness of the DGEBA–Tris changes only by 0–2 nm, and the final thickness is reached within 200 s of dwell.

Adhesion between the surfaces builds up in contact during compression and dwell. During dwell, the contact area increases continuously, consistent with a buildup of adhesive interactions. After 5 min of dwell, the surfaces are pulled apart at a constant drive velocity until detachment, which occurs through a jump-out instability. In N_2_, adhesive failure occurs at less than 3% strain, while in water, the film does not stretch beyond the equilibrium swollen thickness during retraction. Failure appears to be adhesive, but we cannot rule out the possibility of some small transfer to the other surface.^47^ After detachment, we do not observe significant changes either in the surface profile or in the contact region if we make subsequent contact at the same spot, consistent with adhesive failure. We did, however, notice that the pull-off force would decrease if we measured adhesion multiple times at the same spot, likely due to some damage or changes in the surface. Therefore, contact spots were changed for each measurement.

Adhesion in dry N_2_ and water, as characterized by the adhesive strength (*F*_C_, pull-off force), is surprisingly similar (Figure 7b). We utilize the JKR relationship in the limit of an elastic half-space (see Supporting Information Section 6 for applicability) to relate the critical strain energy release rate *G*_c_ to the pull-off force through^48,49^
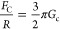
4eq 4 is only valid at the point of adhesive failure where *G* = *G*_c_ by definition. The DGEBA–Tris films investigated here are highly confined, *a*/*h* ∼ 90, and in this limit, eq 4 can lead to errors of up to 30%.^32,50^ We assume that the error is similar across samples because they all have relatively the same degree of confinement as well as comparable glue and mica thicknesses. A complex analysis of the multilayered system compliance would be necessary to precisely correct for confinement in the SFA.^32,50^

**Figure 8:**
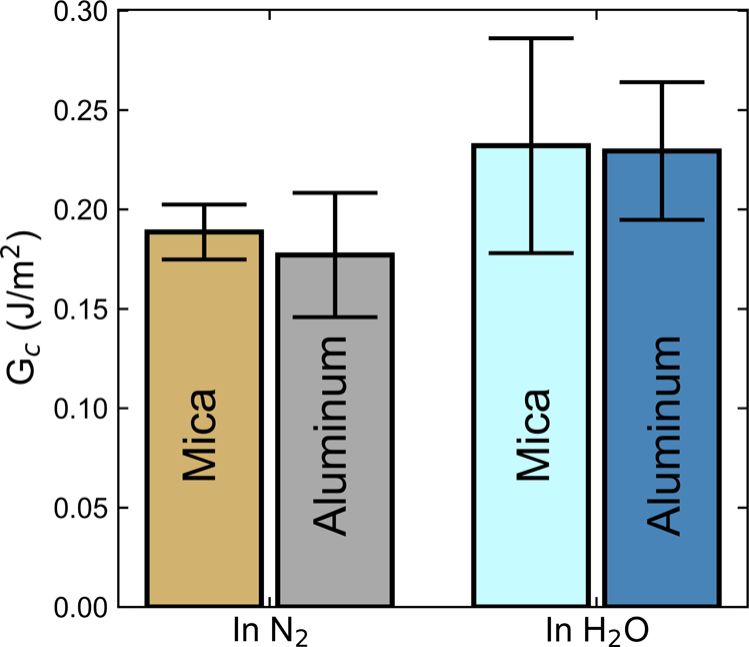
Adhesion of DGEBA–Tris with mica or aluminum in N_2_ and in water. Average critical energy release rate for the adhesion of DGEBA–Tris oligomers to mica or aluminum surfaces in dry N_2_ or in water. No loss of adhesion is seen when contact is made in water. Retraction velocities were 23–35 nm/s for contact with mica and 5–10 nm/s for contact with aluminum.

The adhesion of DGEBA–Tris oligomers in water is as strong as that in dry N_2_ (Figure 8). In N_2_, *G*_c_ = 0.19 ± 0.01 J/m^2^ for contact between DGEBA–Tris and mica, while in water, it is *G*_c_ = 0.23 ± 0.05 J/m^2^. This is remarkable since, typically, underwater adhesion is much weaker than that in air because of lower van der Waals interactions.^51,52^ Surface enrichment of Tris groups in water could help make up for the weakened van der Waals forces, and swelling of the film could facilitate contact area in water. We further investigate the adhesion of DGEBA–Tris oligomers with ultra-smooth aluminum films.^26^ These films have a natural Al_2_O_3_ layer on the surface, which should give them a higher density of hydrogen-bond-accepting groups than mica.^53^ Adhesion of DGEBA–Tris films with aluminum also remains strong underwater with *G*_c_ = 0.18 ± 0.03 J/m^2^ in N_2_ and *G*_c_ = 0.23 ± 0.03 J/m^2^ in water. The similarity in adhesion may indicate that we are more limited by the density of Tris moieties than by hydrogen-bond-accepting groups on the opposing surface.

To investigate the rate-dependent adhesion in water, we extract the instantaneous radial velocity during debonding from the interference fringes. The radial velocity *u*_r_ = −d*a*/d*t* is nearly constant during most of the retraction; then, as we approach the pull-off force, the radial velocity increases rapidly until the surfaces jump out of contact. To obtain *u*_r,c_ at pull-off, we rely on d*a*/d*t* calculated over the last 1 μm change in contact radius. We then obtain an estimate for *G*_0_ by assuming that the only rate-independent interactions are those caused by van der Waals forces (see Supporting Information Section 7). By using the Lifshitz method for estimating non-retarded Hamaker constants,^54^ known parameters for mica and water,^52,54^ and refractive index and dielectric permittivity for the oligomer from ellipsometry measurements and literature,^55^ respectively, we estimate the van der Waals adhesion of DGEBA–Tris oligomer and mica in water to be 12 mJ/m^2^. This estimated *G*_0_ is much lower than the smallest measured *G*_c_ (79 mJ/m^2^). Therefore, an error of *O*(1) on this estimate will not impact the magnitude of the *G*_c_ – *G*_0_ term.

**Figure 9:**
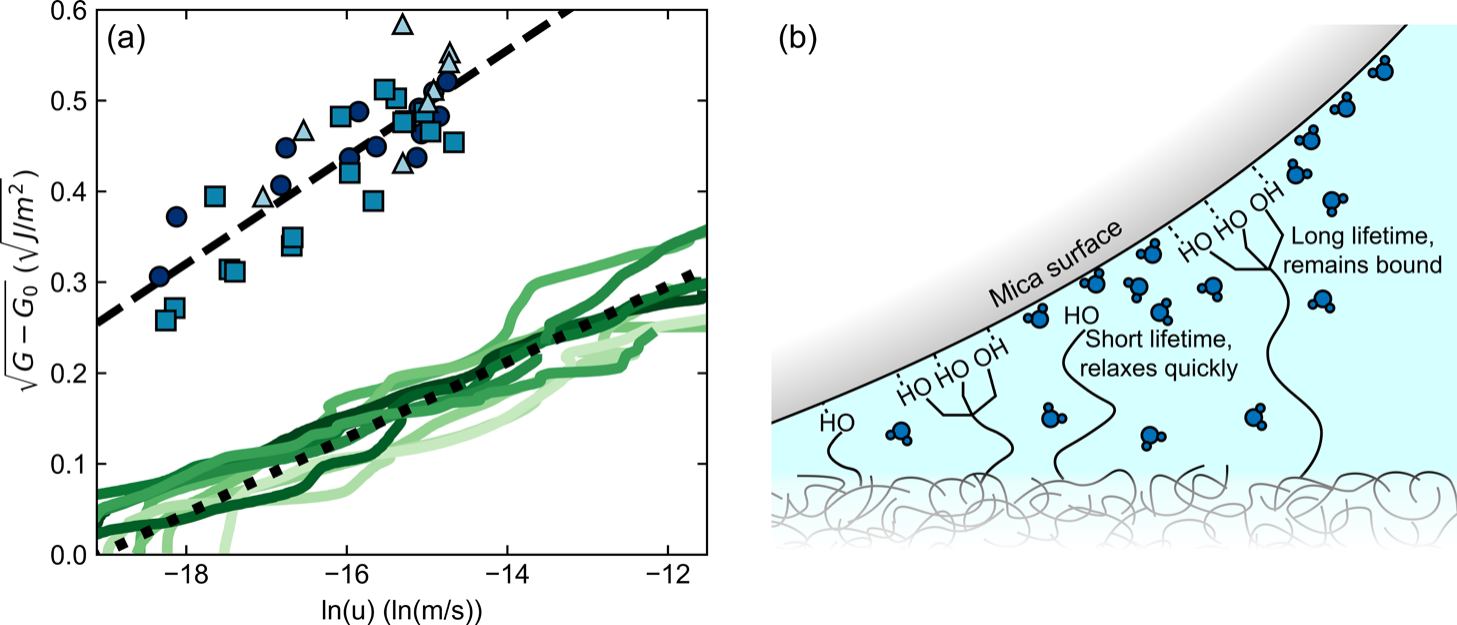
Rate dependence of energy release rate reveals cooperative H-bonding in air and water. (a) Difference between the measured critical strain energy release rate, *G*, and the energy release rate *G*_0_ as a function of the crack velocity (*u*). Interfacial crack propagation water with the SFA is shown as blue symbols (different colors/shapes indicating separate samples), where *G* = *G*_c_ and *u* = *u*_r,c_. Self-arresting crack measurements in air are shown as green lines. The dashed line indicates a fit of measurements in water (*R*^2^ = 0.72) to eq 1, while the dotted line shows the aggregated fit to self-arresting crack measurements in air. (b) Schematic illustrating the proposed cooperative debonding mechanism where cooperative hydrogen bonds have a significantly longer bond lifetime and contribute strongly to underwater adhesion where the adhesive strength depends strongly on crack velocity.

As for measurements in air, adhesion between DGEBA–Tris oligomers and mica is stronger as the crack velocity increases (Figure 9a) and follows the scaling expected for the breaking of interfacial bonds (eq 1). The values of  are larger in water than in air, partially because the weaker van der Waals contributions in water render *G*_o_ an order of magnitude weaker in water. The slope of  vs ln(*u*) is slightly higher in water than in air, and the intercept occurs at a slower velocity. The discrepancy in geometry and errors, for example, due to confinement, in the measurement of *u*_r,c_ and *G*_c_ in the SFA could explain this difference. Given the critical nature of the failure in the SFA, it is more challenging to capture *u*_r,c_ than the stable *u* measured through self-arresting crack propagation. It is also possible that swelling of the epoxy film and surface enrichment of Tris groups could enhance adhesion compared to the dry case and will be the subject of future investigations.

We follow the same approach as for the measurements in air to obtain estimates of the bond lifetime for the adhesion measurements between DGEBA–Tris and mica in water. Using the same limiting values of Σ and *M* listed in Table 1, we estimate the bond lifetime in water to be between0.08 s ≤ τ ≤ 6 s, leading to an equilibrium activation energy of 27*k*_B_*T* ≤ *E*_a_ ≤ 31*k*_B_*T*. Using the Lake–Thomas value of Σ yields, τ = 3 ± 1 s and *E*_a_ = 30.3 ± 0.6*k*_B_*T*. These values, while higher, are remarkably similar to the measurements in air, especially given differences in geometry, environment, and experimental protocol. We thus conclude that similar binding kinetics occur in water and in air, and the values suggest that tridentate H-bonding contributes to adhesion in both cases.

Our observations are in qualitative agreement with prior work on cooperative debonding in adhesion. In particular, several single-molecule studies have obtained lifetimes of *O*(ms) for multidentate hydrogen bonding in Dopa and between UPy groups.^5,6,43^ These force microscopy experiments showed stronger hydrogen-bonding interactions when multiple −OH groups are involved, and that cooperativity substantially increases the lifetime of the bond. Here, we see an analogous effect but in macroscale adhesion measurements.

The proposed cooperative debonding mechanism is illustrated in Figure 9b. The measured adhesive strength and its dependence on crack velocity cannot be obtained by the debonding of a larger number of individual (and independent) hydrogen bonds. One possible explanation as to why the Tris moieties lead to cooperativity could be that anchoring of an individual (or two) −OH group(s) in the Tris moiety restricts the movement of the third group on the surface. The hindered mobility on the surface could encourage rapid rebinding and longer effective bond lifetimes for the whole moiety. A similar mechanism has been suggested by molecular dynamics simulations of Dopa molecules interacting with alumina surfaces in water.^56^ This enhanced binding affinity could help explain the ability of Tris-modified epoxies to maintain their adhesion strength in the presence of water.^10^ For individual hydrogen bonds, interfacial water will compete for surface binding sites and interact with hydroxyl groups in the adhesive,^57−59^ shifting the binding equilibrium and drastically lowering the number of adhesive hydrogen-bonding interactions and thus the total adhesive force of the system.^57,60^ However, if the tridentate bonds are more strongly anchored to the opposing surface, the binding equilibrium would be less disrupted by interfacial water, allowing a higher number of Tris groups to remain bound and maintaining the adhesive strength.^9^ Additionally, computational studies have shown that the adsorption of catechols to a hydrated interface and subsequent displacement of interfacial water is energetically favorable.^61,62^ A similar mechanism could allow Tris groups to form adhesive bonds even in the presence of interfacial water. However, the ability to form tridentate interactions could be strongly dependent on the density of hydrogen-bonding sites on the opposing surface, with less dense surfaces not allowing for three interactions within the reach of the three arms of the Tris moiety.^9^

### Alternative Mechanisms

We also rule out bulk dissipation in the form of viscoelasticity or film stretching as a possible cause for the increase in adhesion with crack velocity. The observed rate dependence occurs at velocities that are slower than those typically observed for bulk viscoelastic dissipation. The widely used semi-empirical model for viscoelastic rate dependence, , attributes an increase in adhesion to dissipation during extension, and thus higher values of *G* are typically correlated with increased extension during detachment.^63,64^ Instead, the opposite trend occurs in our SFA measurements: at higher loading rates, lower extension is seen, even though *G* increases. Overall, we see minimal extension (<8 nm) in our films and therefore expect that bulk dissipation in the film is negligible.

The SFA measurements also allow us to confirm the validity of eq 3 for our peeling measurements. Equation 3 assumes that there is minimal deformation in the film during detachment and that the substrate is rigid. With a similar film thickness, our SFA measurements reveal <8 nm of stretching in all cases, and the fracture appears to be interfacial. Any deformation in the film is clearly minimal in comparison to the bending of the top sheet, and thus the bending can be modeled as the flexure of an elastic plate.

## Conclusions

Adhesion of model epoxy oligomers modified with tridentate hydroxyl groups (DGEBA–Tris) with mica was investigated in air and in water. We showed that the critical strain energy release rate of DGEBA–Tris epoxies scales with the crack velocity according to Chaudhury’s rate-dependent fracture model,^16^ and that control experiments with epoxies that do not contain the Tris moiety showed no dependence on adhesion with crack velocity. Our data suggest that adhesion involving DGEBA–Tris films is due to long-lived interfacial interactions by Tris groups. We also found that the pull-off force of DGEBA–Tris and mica was maintained in the presence of water (*F*/*R* = 0.94 ± 0.3 N/m in water vs 0.89 ± 0.16 N/m in N_2_ for comparable detachment velocity). DGEBA–Tris also showed sustained adhesion with aluminum films in air and water. In addition, adhesion in water also increases with an increase in detachment velocity. By placing conservative limits on molecular parameters, we estimate that the lifetime of these interfacial bonds is between 0.002 and 0.6 s in air and between 0.08 and 6 s in water, corresponding to a bond activation energy of between 23 and 29 *k*_B_*T* in air and between 27 and 31 *k*_B_*T* in water. These lifetimes and activation energies are consistent with three hydrogen bonds working cooperatively to form a single, robustly bonded group that resists displacement by interfacial water. We propose that the enhanced lifetime of this bond is responsible for its strong underwater adhesion. These findings provide quantitative insight into the connection between the molecular physics and macroscale adhesion of epoxy adhesives containing multidentate hydrogen-bonding moieties and suggest a mechanism via which the use of such groups could be used to overcome the detrimental impacts of interfacial water on adhesion in real-world applications.

## Supporting Information Available

Diagram of SFA; variability in the adhesion of cured DGEBA–Tris; estimation of chain density through the Lake–Thomas theory; calculation of oligomer spring constant; analysis of double-layer repulsion; use of JKR equation; estimation of *G*_0_ in water; and analysis of poroelastic flow during retraction (PDF)
