# Supplementary material for: Cooperative Tridentate Hydrogen-Bonding Interactions Enable Strong Underwater Adhesion
Source: ACS Appl Mater Interfaces. 2023 Jul 14;15(29):35720–31. doi: 10.1021/acsami.3c06545 (PMC10375471; doi:10.1021/acsami.3c06545)
Supplement: Supplementary file 1 — am3c06545_si_001.pdf [file am3c06545_si_001.pdf]

Supporting Information

Cooperative Tridentate Hydrogen Bonding Interactions Enable Strong Underwater Adhesion

Zachary D. Lamberty[1], Ngon T. Tran[2], Christian D. van Engers[3], Preetika Karnal[4], Daniel B. Knorr, Jr.[2], and Joelle Frechette,[1*]*

1. Chemical and Biomolecular Engineering Department, University of California, Berkeley, Berkeley, California, 94760, USA.

2. DEVCOM U.S. Army Research Laboratory, Aberdeen Proving Ground, Maryland 21005, USA.

3. Department of Chemical and Biomolecular Engineering, Johns Hopkins University, Baltimore, Maryland, 21218, USA.

Current address: *School of Science, Royal Melbourne Institute of Technology, Melbourne, Victoria 3000, Australia*

4. Department of Chemical and Biomolecular Engineering, Lehigh University, 124 E Morton St, Building 205, Bethlehem, Pennsylvania 18015, USA

***Corresponding authors:**

Joelle Frechette jfrechette@berkeley.edu and Daniel B. Knorr, Jr: daniel.b.knorr.civ@army.mil

Contents

S1. Diagram of Surface Forces Apparatus (SFA) 2

S2. Variability in adhesion of cured DGEBA-Tris 2

S3. Calculation of oligomeric spring constant 3

S4. Estimation of chain density through Lake-Thomas Theory 4

S5. Analysis of double layer repulsion. 4

S6. Use of JKR equation. 5

S7. Estimation of G_0 in water 5

S8. Poroelastic flow during retraction 6

References 7

S1. Diagram of Surface Forces Apparatus (SFA)

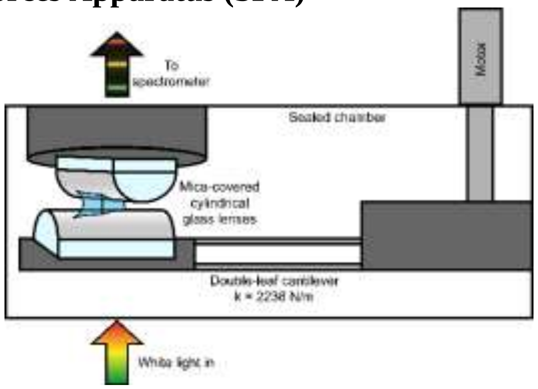

Figure S1. Diagram of SFA. Diagram detailing the configuration of the SFA used for these measurements. One hemi-cylindrical lens is mounted on a double-leaf cantilever, while the other is fixed to a stationary top mount. A microstepping motor allows for positioning of the bottom lens to apply forces. A water drop is injected between the surface to form a capillary and submerge the contact region. Extensive diagrams of the SFA can be found elsewhere in literature.[1] Diagram is not to scale.

S2. Variability in adhesion of cured DGEBA-Tris

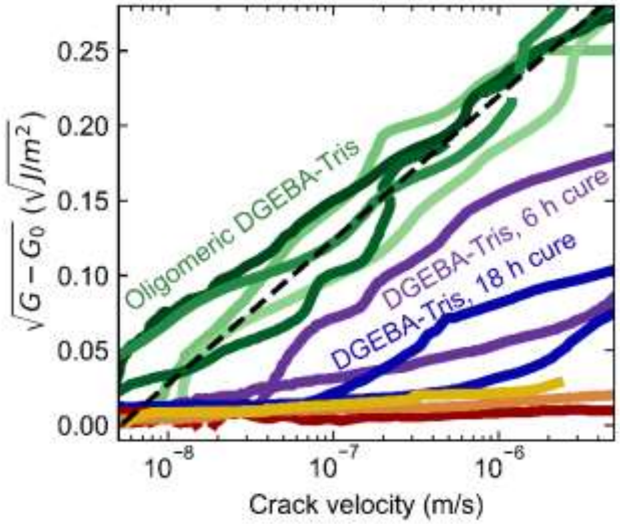

Figure S2. Variability in adhesion dynamics. Scaled energy release rate vs crack velocity for bare H^+ mica (orange), oligomeric DGEBA-Tris (green), DGEBA-Tris cured for 6 h (purple) or 18 h at 150 ºC (blue), and DGEBA-355 (yellow) and DGEBA-1750 (red), both cured for 18 h at 150 ºC. Cured DGEBA-Tris reliably exhibits enhancement of adhesion with crack velocity with a higher onset velocity than for oligomeric DGEBA-Tris.

S3. Calculation of oligomeric spring constant

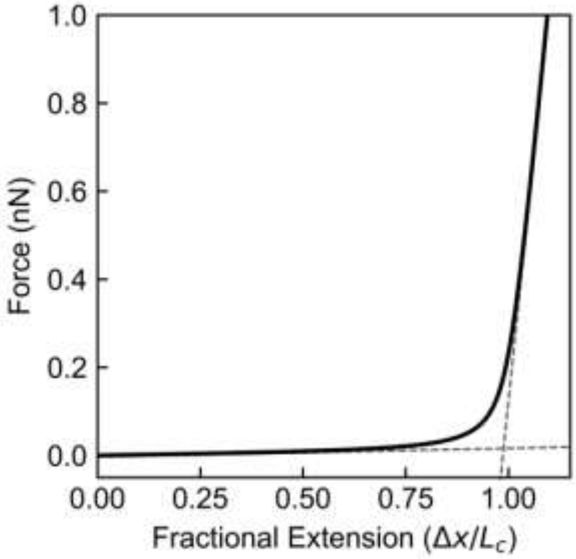

Figure S3. Estimated force-extension relationship for a DGEBA-Tris oligomer. Force vs estimated fractional extension ($\Delta x/L_c$) for a DGEBA-Tris oligomer, using **Eqn. S1** with $l_k \approx$ 0.78 nm, n = 2.83, and $K_{total} \approx$ 5 N/m. The lower asymptote corresponds to a minimum entropic spring constant of 8 mN/m, while large extension is dominated by bond deformation, giving an upper limit of K_{total}.

In order to estimate the force-extension relationship for the complex DGEBA-Tris oligomer, we compare segments of the DGEBA-Tris molecule to the stretching behavior of chains in literature. While several models for the force-extension relationship of a polymer exist,[2] we choose to use the modified-Freely Jointed Chain (m-FJC) model due to plethora of literature employing this model and reporting parameters for various polymer chemistries. The m-FJC is given by:[2]

$$\Delta x = \left[\coth\left(\frac{Fl_k}{k_BT}\right) - \frac{k_BT}{Fl_k}\right]\left(L_c + \frac{nF}{K_{segment}}\right). \qquad \text{(S1)}$$

In this model Δx is the extension of the chain, l_k is the Kuhn length of each segment in the chain, L_c is the contour length of the chain, n is the number of Kuhn segments, and $K_{segment}$ is the stiffness of each segment. The aromatic section of the DGEBA molecule can be estimated to have $l_k \approx$ 0.87 nm and $K_{aromatic} \approx$ 960 N/m by comparison to the stretching similar polymers in literature.[3] The linear aliphatic section of the DGEBA-Tris molecule is estimated to have $l_k \approx$ 0.72 nm and $K_{aliphatic} \approx$ 7.6 N/m by similar comparison to alkane and PEG chains in literature.[4-7]. A simple weighted average approximates the chain as 2.52 segments of $l_k \approx$ 0.78 nm, giving L_c = 1.97 nm, and adding the stiffness of each segment in series gives $K_{total} \approx$ 5 N/m. Using these values, the estimated force-extension relationship of the oligomer is shown in **Fig. S3**. At low extension, the oligomer is predicted to stretch entropically with a minimum spring constant (dF/dx) of 8 mN/m. At large values fractional extension, the deformation of bonds becomes significant with the spring constant asymptotically approaching $K_{total} \approx$ 5 N/m. The effective spring constant M of the oligomer must fall between these limiting values. We note that the prediction *a priori* of a force-extension relationship, especially for a complex oligomer, is challenging. However, these

limits represent extreme conditions, and the estimates of M from other studies give values on O 0.1 N/m, well within our range.

S4. Estimation of chain density through Lake-Thomas Theory

Lake-Thomas theory states that the number of chains crossing a plane can be calculated through[8]

$$\frac{\text{\# of chains}}{\text{unit area}} = \frac{1}{2}\sqrt{\frac{8n}{3\pi}}\, l_k N. \tag{S2}$$

Here N is the chain number density and l_k and n are the Kuhn length and number of Kuhn segments, respectively, for the chain. We approximate that $\Sigma \approx \frac{\text{\# of chains}}{\text{unit area}}$ at the fracture plane, and then use the values of l_k and n found in **Section S3** with $N \approx 8.72\text{x}10^{26}$ chains/m^3 to obtain $\Sigma \approx$ 5.0x10^{17} Tris groups/m^2.[9] This assumes that all Tris groups at the interface are bound and thus likely overestimates Σ, which therefore underestimates τ and E_a. As we are attempting to ascertain if E_a is of $O(10k_BT)$ or of $O(30k_BT)$, underestimating E_a to be between $23\ k_BT$ - $31\ k_BT$ does not alter our conclusions.

S5. Analysis of double layer repulsion.

In SFA experiments when samples are brought together in water a long-range repulsive force is measured before contact is made. These long-range forces measured at separation > 80 nm are consistent with double layer repulsion. The force decays exponentially with an increase in surface separation (**Fig. 7a**) and a Debye length of κ^{-1} = 24 $\pm$ 3 nm. The ionic concentration of the solution can be estimated from the Debye length as 1.6x10^{-4} $\pm$ 0.2x10^{-4} M, assuming a symmetric 1:1 electrolyte.[10] This is a higher concentration than the ~ 10^{-5} M expected for DI water in equilibrium with atmospheric CO_2, suggesting that there could be additional ionic species from sample preparation steps or the DI water or multivalent ions present. We can also compare our data to the force expected for the interaction between electrostatic double layers using the non-linear superposition and the Derjaguin approximation: [10]

$$\frac{F}{R} = \kappa\left[64\pi\epsilon\epsilon_0\left(\frac{k_BT}{e_c}\right)^2 \tanh\left(\frac{e_c\,\psi_{mica}}{4k_BT}\right)\tanh\left(\frac{e_c\,\psi_{DGEBA-tris}}{4k_BT}\right)\right]\exp(-\kappa D). \tag{S3}$$

Here F is the force, R is the radius of curvature of the sample, D is the separation between the surfaces, e_c is the elementary charge, $\epsilon\epsilon_0$ is the dielectric permittivity of the medium (i.e. water), and ψ_{mica} and ψ_{tris} are the surface potentials of the mica and DGEBA-Tris surfaces, respectively. By using a previously measured value of $\psi_{mica} = -150$ mV,[11] we estimate the surface potential of the DGEBA-Tris film to be $\psi_{DGEBA-tris} = -31 \pm 5$ mV.

S6. Use of JKR equation.

The Johnson-Kendall-Roberts (JKR) equation relates the force to the contact area for in the contact of a sphere-on-flat geometry.[12]

$$a^3 = \frac{3R}{4E^*}\left(F + 3\pi GR + (6\pi GRF + (3\pi GR)^2)^{\frac{1}{2}}\right). \quad (S4)$$

Here a is the contact radius, F is the force, R is the radius of curvature, E^* is the reduced elastic modulus of the sample, and G is the strain energy release rate. The JKR relation is applicable in the limit of soft, large radius of curvature, and highly adhesive materials that don't interact outside of contact. Both Tabor and Maugis developed parameters describing the transition from the JKR limit to the opposing regime, described by the Derjaguin-Muller-Toporov (DMT) equation.[13] The Tabor parameter is given by:[14]

$$\mu \equiv \left(\frac{RG^2}{{E^*}^2 z_0^3}\right)^{\frac{1}{3}}, \quad (S5)$$

where z_0 is the equilibrium surface separation. If $\mu \gg 1$, the samples will be in the JKR limit. With z_0 estimated as 0.2 nm, and effective modulus of the system $E^* = 6$ GPa,[14], and our lowest adhesion energy of $G = 79$ mJ/m^2, $\mu = 69$. Even with $z_0 = 1$ nm, the RMS roughness of our DGEBA-Tris films after 24 hours in water, $\mu = 14$ and the condition is still satisfied.

The Maugis parameter is given by:[13]

$$\lambda \equiv \frac{2\sigma_0}{\left(\frac{16\pi G{E^*}^2}{9R}\right)^{\frac{1}{3}}}, \quad (S6)$$

where σ_0 is the yield stress of the material. The JKR limit is reached as $\lambda \rightarrow \infty$, but values of $\lambda > 5$ give only small deviations from the JKR equation.[13] If we approximate $\sigma_0 \approx 1.03\frac{G}{z_0}$,[13] the previously used values of z_0 give $\lambda = 80$ and $\lambda = 16$, respectively. Both values are indicative of JKR-like mechanics.

The final assumption of the JKR equation, that there is no interaction outside of the contact region, is violated by the electrostatic repulsive forces that we measure on approach (*c. f.* **Fig. 7** in the main text). However, as these forces are at a maximum $\frac{F_{electrostatic}}{R} \approx 1$ mN/m, while the adhesive forces are at minimum 370 mN/m at pull-off, we expect deviation from JKR due to electrostatic repulsion to be small.

S7. Estimation of G_0 in water

To estimate the rate-independent strain energy release rate in water we first assume that all rate-independent interactions are due to van der Waals forces. In this system, interactions of DGEBA-Tris with mica across water as well as the interaction of the underlying mica substrate with water across the thin DGEBA-Tris film. The energy of this interaction is given by:[15]

$$G_{vdW} = -\frac{1}{12\pi}\left[\frac{A_{132}}{D^2} - \frac{A_{213}}{(D+H)^2}\right]. \quad (S7)$$

In this equation, A_{132} is the Hamaker constant of material 1 interacting with material 2 across medium 3, where 1 is DGEBA-Tris, 2 is mica, and 3 is water. D is the separation between the DGEBA-Tris film and the opposing mica surface, while H is the thickness of the DGEBA-Tris film. The Hamaker constants can be estimated using Lifshitz theory.[15]

$$A_{132} \approx \frac{3}{4} k_B T \left(\frac{\varepsilon_1-\varepsilon_3}{\varepsilon_1+\varepsilon_3}\right)\left(\frac{\varepsilon_2-\varepsilon_3}{\varepsilon_2+\varepsilon_3}\right) + \frac{3h\nu_e}{8\sqrt{2}} \frac{(n_1^2-n_3^2)(n_2^2-n_3^2)}{(n_1^2+n_3^2)^{\frac{1}{2}}(n_2^2+n_3^2)^{\frac{1}{2}}\left[(n_1^2+n_3^2)^{\frac{1}{2}}+(n_2^2+n_3^2)^{\frac{1}{2}}\right]} \quad \text{(S8)}$$

In this equation, k_B is Boltzmann's constant, T is the temperature (20 °C), ε_1 is the dielectric permittivity of material 1 (DGEBA-Tris), h is Planck's constant, ν_e is the electronic absorption frequency (assumed to be 3×10^{15} s^{-1}), and n_1 is the refractive index of material 1. We use literature values for the dielectric permittivity of mica and water,[15, 16] the measured value of the refractive index of DGEBA-Tris (see methods section in main text), and an estimate of the dielectric permittivity of DGEBA-Tris from literature.[17] These parameters are summarized in **Table S1.** For a contact $D = 0.2$ nm, **Eqns. S7** and **S8** estimate that $G_{vdW} \approx$ 12 mJ/m^2.

Table S1. Parameters used in Eqn. S6 and S7 to calculate G_{vdW}.

T	ν_e [b]	ε_1 [a]	ε_2 [b]	ε_3 [b]	n_1	n_2 [b]	n_3 [b]	A_{132}	A_{213}	H
°C	s^{-1}							J	J	nm
20	3×10^{15}	16	6.2	80	1.582	1.60	1.333	1.85×10^{-20}	0.23×10^{-20}	70

(a) From [17], (b) from [15].

S8. Poroelastic flow during retraction.

Following the procedure of Delavoipiere *et al.,* the force due to fluid flow into or out of a porous material during compression or extension can be modeled as:

$$F_H = \frac{\pi}{8H}\left(\frac{\dot{\delta}\eta a^4}{\kappa}\right) \quad \text{(S9)}$$

Here F is the force on the indenter, a is the contact radius, δ is the indentation depth, $\dot{\delta}$ is the indentation rate, H is the film thickness, η is the dynamic viscosity of the solvent, and κ is the permeability of the film. By using measured values of a and $\dot{\delta}$, we can estimate the force due to fluid infusion for various values of η/κ and compare them to the measured forces at various retraction velocities. **Fig S4** shows that for commonly used values of η/κ (purple, $\eta = 8.9 \times 10^{-4}$ Pa s and $\kappa = 1$ nm^2),[18] the estimated force due to fluid infusion is negligible. Only when η/κ is increased by over 6 orders of magnitude (orange, $\frac{\eta}{\kappa} = 1.8 \times 10^{21}$ Pa s/m^2) is the force significant in comparison to the measured forces (blue, green, and red points), and decreases sharply near pull-off as a decreases. Qualitatively, our measured forces do not agree with this model, and the unreasonable values of η/κ needed to produce forces in the range of our measurements indicates that fluid infusion is unlikely to affect our measured forces significantly.

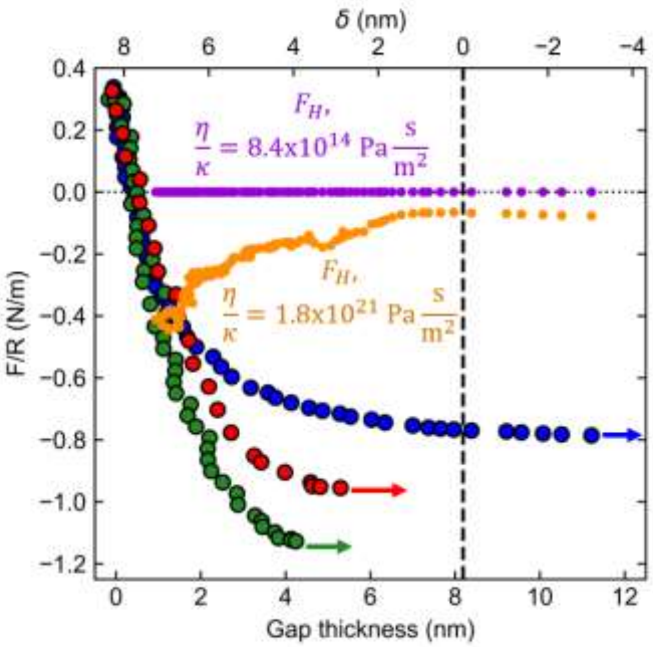

Figure S4. Estimated fluid-infusion force during retraction in water. Force normalized by radius of curvature for estimated force due to fluid infusion during retraction using **Eqn. S9**, with literature values for $\frac{\eta}{\kappa} = 8.4x10^{14}$ Pa s/m^2 (purple) and extreme values needed to model indentation curve $\frac{\eta}{\kappa} = 1.8x10^{21}$ Pa s/m^2 (orange). For $\delta > 7.2$ nm (Gap thickness < 1 nm), $\dot{\delta}$ is below our measurement sensitivity and is therefore not plotted. Data is overlayed onto the measured curves for the three different retraction velocities with V_{motor} = 11 nm/s (blue), V_{motor} = 24 nm/s (red), and V_{motor} = 71 nm/s (green), to facilitate comparison. Even if unreasonable values of η/κ are used, the estimated porous infusion force decays near pull-off due to sharp decreases in a.

References

(1) Israelachvili, J.; Min, Y.; Akbulut, M.; Alig, A.; Carver, G.; Greene, W.; Kristiansen, K.; Meyer, E.; Pesika, N.; Rosenberg, K.; et al. Recent Advances in the Surface Forces Apparatus (SFA) Technique. *Rep. Prog. Phys.* **2010**, *73* (3). DOI: 10.1088/0034-4885/73/3/036601

(2) Giannotti, M. I.; Vancso, G. J. Interrogation of Single Synthetic Polymer Chains and Polysaccharides by AFM-Based Force Spectroscopy. *Chemphyschem* **2007**, *8* (16), 2290-2307. DOI: 10.1002/cphc.200700175

(3) Yu, Y.; Zhang, Y.; Jiang, Z.; Zhang, X.; Zhang, H.; Wang, X. Full View of Single-Molecule Force Spectroscopy of Polyaniline in Oxidized, Reduced, and Doped States. *Langmuir* **2009**, *25* (17), 10002-10006. DOI: 10.1021/la901169p

(4) Chen, J.; Wu, M.; Gong, L.; Zhang, J.; Yan, B.; Liu, J.; Zhang, H.; Thundat, T.; Zeng, H. Mechanistic Understanding and Nanomechanics of Multiple Hydrogen-Bonding Interactions in Aqueous Environment. *J. Phys. Chem. C* **2019**, *123* (7), 4540-4548. DOI: 10.1021/acs.jpcc.8b11790

(5) Zou, S.; Schönherr, H.; Vancso, G. J. Stretching and Rupturing Individual Supramolecular Polymer Chains by AFM. *Angew. Chem.* **2005**, *117* (6), 978-981. DOI: 10.1002/ange.200460963

(6) Wang, S.; Panyukov, S.; Rubinstein, M.; Craig, S. L. Quantitative Adjustment to the Molecular Energy Parameter in the Lake–Thomas Theory of Polymer Fracture Energy. *Macromolecules* **2019**, *52* (7), 2772-2777. DOI: 10.1021/acs.macromol.8b02341

(7) Li, H.; Liu, B.; Zhang, X.; Gao, C.; Shen, J.; Zou, G. Single-Molecule Force Spectroscopy on Poly(Acrylic Acid) by AFM. *Langmuir* **1999**, *15* (6), 2120-2124. DOI: 10.1021/la9800304

(8) Lake, G. J.; Thomas, A. G. The Strength of Highly Elastic Materials. *Proc. R. Soc. London, Ser. A* **1967**, *300* (1460), 108-119. DOI: 10.1098/rspa.1967.0160

(9) McAninch, I. M.; Palmese, G. R.; Lenhart, J. L.; La Scala, J. J. Epoxy-Amine Networks with Varying Epoxy Polydispersity. *J. Appl. Poly. Sci.* **2015**, *132* (8), n/a-n/a. DOI: 10.1002/app.41503

(10) Israelachvili, J. *Intermolecular and Surface Forces*; Academic Press, 2011, pp 291-337.

(11) van Engers, C. D.; Lamberty, Z. D.; McGuiggan, P. M.; Frechette, J. Template-Stripped Ultra-Smooth Aluminum Films (0.2 Nm Rms) for the Surface Forces Apparatus. *Langmuir* **2021**, *37* (21), 6556-6565. DOI: 10.1021/acs.langmuir.1c00899

(12) Johnson, K. L.; Kendall, K.; Roberts, A. D. Surface Energy and the Contact of Elastic Solids. *Proc. R. Soc. London, Ser. A* **1971**, *324*, 301-313. DOI: 10.1098/rspa.1971.0141

(13) Maugis, D. Adhesion of Spheres: The JKR-Dmt Transition Using a Dugdale Model. *J. Colloid Interface Sci.* **1992**, *150* (1), 243-269. DOI: 10.1016/0021-9797(92)90285-t

(14) McGuiggan, P. M.; Wallace, J. S.; Smith, D. T.; Sridhar, I.; Zheng, Z. W.; Johnson, K. L. Contact Mechanics of Layered Elastic Materials: Experiment and Theory. *J. Phys. D: Appl. Phys.* **2007**, *40* (19), 5984-5994. DOI: 10.1088/0022-3727/40/19/031

(15) Israelachvili, J. *Intermolecular and Surface Forces*; Academic Press, 2011, pp 253-284.

(16) Christenson, H. K. Adhesion and Surface Energy of Mica in Air and Water. *J. Polym. Chem.* **1993**, *97*, 12034-12041.

(17) Sheppard, N. F.; Senturia, S. D. Chemical Interpretation of the Relaxed Permittivity During Epoxy Resin Cure. *Polymer Engineering and Science* **1986**, *26* (5), 354-357. DOI: 10.1002/pen.760260505

(18) Degen, G. D.; Chen, Y. T.; Chau, A. L.; Mansson, L. K.; Pitenis, A. A. Poroelasticity of Highly Confined Hydrogel Films Measured with a Surface Forces Apparatus. *Soft Matter* **2020**, *16* (35), 8096-8100. DOI: 10.1039/d0sm01312a
